# Herbivory of an invasive slug is affected by earthworms and the composition of plant communities

**DOI:** 10.1186/1472-6785-13-20

**Published:** 2013-05-13

**Authors:** Johann G Zaller, Myriam Parth, Ilona Szunyogh, Ines Semmelrock, Susanne Sochurek, Marcia Pinheiro, Thomas Frank, Thomas Drapela

**Affiliations:** 1 Institute of Zoology, Department of Integrative Biology and Biodiversity Research, University of Natural Resources and Life Sciences Vienna, Gregor Mendel Straße 33, Vienna, A-1180, Austria; 2 Research Institute of Organic Agriculture (FiBL Austria), Vienna, A-1070, Austria

**Keywords:** Belowground-aboveground interactions, Ecosystem functioning, Biodiversity loss, Plant-animal interactions, Soil invertebrates, Invasive herbivores, Plant community composition, Global change ecology

## Abstract

**Background:**

Biodiversity loss and species invasions are among the most important human-induced global changes. Moreover, these two processes are interlinked as ecosystem invasibility is considered to increase with decreasing biodiversity. In temperate grasslands, earthworms serve as important ecosystem engineers making up the majority of soil faunal biomass. Herbivore behaviour has been shown to be affected by earthworms, however it is unclear whether these effects differ with the composition of plant communities. To test this we conducted a mesocosm experiment where we added earthworms (Annelida: Lumbricidae) to planted grassland communities with different plant species composition (3 vs. 12 plant spp.). Plant communities had equal plant densities and ratios of the functional groups grasses, non-leguminous forbs and legumes. Later, *Arion vulgaris* slugs (formerly known as *A. lusitanicus;* Gastropoda: Arionidae) were added and allowed to freely choose among the available plant species. This slug species is listed among the 100 worst alien species in Europe. We hypothesized that (i) the food choice of slugs would be altered by earthworms’ specific effects on the growth and nutrient content of plant species, (ii) slug herbivory will be less affected by earthworms in plant communities containing more plant species than in those with fewer plant species because of a more readily utilization of plant resources making the impacts of earthworms less pronounced.

**Results:**

Slug herbivory was significantly affected by both earthworms and plant species composition. Slugs damaged 60% less leaves when earthworms were present, regardless of the species composition of the plant communities. Percent leaf area consumed by slugs was 40% lower in communities containing 12 plant species; in communities containing only three species earthworms increased slug leaf area consumption. Grasses were generally avoided by slugs. Leaf length and number of tillers was increased in mesocosms containing more plant species but little influenced by earthworms. Overall shoot biomass was decreased, root biomass increased in plant communities with more plant species. Earthworms decreased total shoot biomass in mesocosms with more plant species but did not affect biomass production of individual functional groups. Plant nitrogen concentrations across three focus species were 18% higher when earthworms were present; composition of plant communities did not affect plant quality.

**Conclusions:**

Given the important role that both herbivores and earthworms play in structuring plant communities the implications of belowground-aboveground linkages should more broadly be considered when investigating global change effects on ecosystems.

## Background

The loss of biodiversity and the invasion by non-native species are among the most important human-induced global change factors [1]. Both processes have generated concern regarding their consequences for ecosystem functioning and understanding the relationship between both has become a major focus in modern ecological research [2-4]. The biodiversity-invasibility hypothesis [5,6] states, that high diversity increases the competitive environment of communities making them harder to invade. Numerous biodiversity experiments have been conducted aiming to test this hypothesis [7-9]. Underlying mechanisms comprise a decreased chance of empty ecological niches, an increased probability of competitors that preclude invasion success, a more complete resource use in diverse communities and limited ability of invaders to establish [4,6,10,11]. Most studies focus on the invasibility of plant communities for invasive plant species [12], however very little is known on the invasibility of plant communities for invasive herbivores. To the best of our knowledge, nothing is known about the role of soil fauna in affecting invasive herbivores in plant communities with different plant species composition.

Earthworms often make up the majority of soil faunal biomass in grasslands, comprise the dominant group of the decomposer community, stimulating the microbial activity and the availability of nutrients in soil [13-15]. In temperate grasslands, these earthworm communities commonly consist of a few species [16] comprising three functional groups: surface dwelling epigeics, vertically burrowing anecics and horizontally burrowing endogeics [17]. The loss of plant species diversity in grasslands has been shown to affect abundance, biomass and activity of earthworms [18-21]. On the other hand, effects of earthworms have been shown to benefit certain plant functional groups more than others [22-25] affecting plant community structure and diversity [26]. The role of earthworms has been most often studied on a single plant species, while only few have investigated effects on plant communities [27-31].

Gastropods (slugs and snails) influence the species composition and relative abundance of plant communities by selectively grazing certain plant species [32-38]. Slug grazing has been shown to shift plant competition in mixed swards and influencing the uptake and partitioning of nutrients among plants [39]. Across Europe, the slug *Arion vulgaris* Moquin-Tandon, formerly known as *A. lusitanicus* is an up to 15 cm long, polyphagous slug species, natively distributed in northern Spain, western France and southern England. This slug is highly invasive all over Europe during the last 30 years and listed among the 100 worst alien species in Europe (http://www.europe-aliens.org/); since 1998 it is also becoming established in the USA [40]. Earthworms have been shown to affect sap-sucking and chewing herbivores such as snails and slugs [33,41] in different ways via changes in leaf chemistry see literature reviewed by [42].

The aim of this study was to test whether (i) slug herbivory in plant communities is affected by earthworms and (ii) whether potential influences of earthworms are altered by the composition of plant communities. We hypothesized that (i) the food choice of slugs would be altered by earthworms’ specific effects on the growth and nutrient content of plant species, (ii) slug herbivory will be less affected by earthworms in plant communities containing more plant species because of a more readily utilization of resources by plants making the impacts of earthworms less pronounced. Slug herbivory on focus species could additionally be affected by potential differences in available plant biomass or microclimate between plant communities differing plant species numbers. These hypotheses were tested in a full-factorial greenhouse experiment using 20-l mesocosms manipulating both earthworms and plant species composition.

## Methods

### Experimental setup

We set up a full-factorial experiment using 20-l mesocosms (diameter 31 cm, height 34 cm) in an unheated greenhouse of the University of Natural Resources and Life Sciences (BOKU), Vienna, Austria. Treatment factors were earthworms (“-Ew” - no earthworms; “+Ew” - addition of two adult individuals of the vertical-burrowing anecic *Lumbricus terrestris* L. and four adult/sub-adult individuals of the soil dwelling endogeic *A. caliginosa* Savigny per mesocosm) and plant community composition (“low diversity” – 3 plant species, “high diversity” – 12 plant species; Table 1). These species are typically co-occurring in many Central European grasslands.

**Table 1:** Plant species composition (# of planted individuals in the community) of mesocosms with low (3 spp.) and high (12 spp.) plant diversity

**Functional type/species**	**Number of individulas planted per treatment**	
	**Low diversity**	**High diversity**
Grasses		
*Arrhenatherum elatius* L.	20	5
*Bromus erectus* Huds.	0	5
*Festuca ovina* L	0	5
*Holcus lanatus* L.	0	5
Forbs		
*Knautia arvensis* L.	0	5
*Leucanthemum ircutianum* Mill.	0	5
*Prunella vulgaris* L.	20	5
*Salvia pratensis* L.	0	5
Legumes		
*Anthyllis vulneraria* L	0	5
*Lotus corniculatus* L	0	5
*Trifolium pratense* L.	20	5
*Vicia cracca* L.	0	5

Planting of mesocosms followed a consistent hexagonal pattern with 60 positions in equidistance of 3.5 cm between plant individuals and the same positions of the functional groups (grasses, forbs and legumes) across all mesocosms. Sowing was done directly in the mescosms by placing two seeds per position; surplus seedlings were removed to achieve the desired planting pattern of 60 plants per mesocosm. The mesocosms were watered with tap water using the same amount for each mesocosm depending on the temperature conditions in the greenhouse. Before we added the earthworms the plants were fertilized once with 20 ml pot^-1^ of customary NPK (7+3+6) fertilizer to foster seedling establishment.

Earthworms were added four weeks after sowing the plants (mean fresh mass ± SE of 8.86 ± 0.13 g mesocosms^-1^*L. terrestris* and 3.16 ± 0.06 g mesocosm^-1^ for *A. caliginosa*); added total earthworm density or biomass translates to 333 individuals m^-2^ or 160 g biomass m^-2^, respectively). All individuals of *A. caliginosa* were collected by hand digging in a garden soil near the city of Wiener Neustadt (Lower Austria), while *L. terrestris* was obtained from a fishing bait shop in Vienna. Mesocosms were lined with plastic fleece at the bottom to prevent earthworms from escaping and then filled with a 40:60% vol/vol field soil:quartz sand substrate mixture (pH-H_2_O = 7.4, C_org_ = 24.2 g kg^-1^, N_tot_ = 0.89 g kg^-1^, P-CAL = 61.1 mg kg^-1^, K-CAL = 107.6 mg kg^-1^). The field soil was a Haplic Chernozem (silty loam) obtained from the BOKU Experimental Farm in Groß-Enzersdorf, near Vienna. This substrate mixture was successfully used in previous studies involving the same earthworm and/or plant species [43,44].

Each treatment was replicated six times. The experiment ran for 12 weeks from April until June 2010. Mean daily air temperature and mean relative humidity in the greenhouse during the experiment was 22.3 ± 0.1°C and 61.3 ± 0.3%, respectively.

Five weeks after adding the earthworms we introduced two sub-adult, 5 cm long specimens of the invasive slug *A. vulgaris* into the specific mesocosms (3.81 g mesocosm^-1^ total slug fresh mass); slugs were collected in the same garden as *A. caliginosa*. Prior to introduction into the mesocosms, slugs were kept in plastic boxes containing field soil in a climate chamber for about one week (10°C, darkness for 24 hours) and given green lettuce *ad libitum*. Feeding with lettuce stopped one day before introducing them into the pots.

To prevent earthworms and/or slugs from escaping out of the pots a 50 cm high transparent plastic film smeared with soft soap at the upper rim was wrapped around each mesocosm. This plastic barrier was also attached to control treatments to provide similar microclimatic conditions across treatments.

### Harvesting procedure and measurements

Earthworm activity was monitored once a week by observing surface casts, burrows or other relevant signs of their activity.

Slug herbivory was measured three times during the course of the experiment. First, three days after slug introduction we counted all leaves growing in the herbivory mesocosms with clear signs of slug damage (lesions). Second, we assessed slug herbivory when harvesting the plants by counting the number of leaves per plant species with slug damage. Third, we measured the leaf area eaten by slugs on scanned leaves of all species (flatbed scanner, 300 dpi resolution) using the freely available image analysis software ImageJ (http://rsbweb.nih.gov/ij/). These measures of slug herbivory were analysed for the three plant species that were present in both plant communities (the forb *P. vulgaris*, the legume *T. pratense* and the grass *A. elatius*.) as well as for the additional species. Seven days after introducing the slugs, they were removed, counted, weighed. Harvesting started by cutting individual plant specimens at the soil surface, measuring maximum plant length and counting number of tillers on three individuals of the grass *A. elatius*, the forb *P. vulgaris* and the legume *T. pratense* mesocosm^-1^. All plant material was dried at 55°C for 48 hours and weighed to assess dry matter production. One individual of the dried focus plant was ground in a ball mill and their C and N concentrations analysed using elementary analysis (LECO-2000, St. Joseph, Michigan, USA).

Belowground biomass was harvested by flipping over the mesocosms and sorting out all roots and earthworms for a period of seven minutes mesocosm^-1^. Earthworms were counted and their fresh masses weighed after rinsing them under tap water and drying them on a paper towel. Roots were washed free of attached soil under a jet of tap water, dried at 55°C for at least three days and weighed.

### Statistical analyses

We tested all variables for homogeneity of variances and normality using the tests after Levene and Kolmogorov-Smirnov, respectively [45]. Assumptions for parametric tests were fulfilled by all tested parameters. Treatment effects were first analysed using two-way ANOVAs with earthworms and plant community composition as fixed factors followed by Tukey post-hoc mean comparisons [45]. We used Pearson correlations to test the relationship between plant nutrient concentrations and slug damage. All statistical tests were performed using SPSS Statistics (vers. 17.0.0., SPSS Inc. Headquarters, Chicago, Illinois, USA). Values given throughout the text are means ± standard error (n = 6).

## Results

We observed earthworm activity throughout the course of the experiment in the form of surface casting, burrow openings or run-over toothpicks that marked plant seedlings (data not shown). At the harvest we found 62% of initially added number of earthworms weighing 42% of the initial earthworm biomass and 90% of the introduced slug individuals weighing 85% of the initial biomass. Earthworm numbers (average between initially added and recovered individuals at harvest) were marginally affected by plant community composition with lower numbers when fewer plant species were present (F_1,20_ = 3.270, P = 0.086) but not affected by slug herbivory. Earthworm biomass (average between initially added biomass and recovered biomass at harvest) was significantly affected by the composition of plant communities with lower biomass when fewer plant species were present (F_1,20_ = 5.949, P = 0.024) but not affected by slug herbivory (data not shown).

Leaf length and number of tillers of *A. elatius* was unaffected by earthworms or the composition of plant communities (Table 2, Table 3). Leaf length and number of tillers of *P. vulgaris* was significantly higher in mesocosms containing more plant species but not affected by earthworms (Table 2, Table 3). Leaf length of *T. pratense* was significantly influenced by earthworms and plant community composition; number of tillers was significantly affected only by plant community composition (significant ew × plant composition interaction; Table 3).

**Table 2:** Leaf length and number of tillers in response to earthworms (−Ew…no earthworms, +Ew…earthworm addition) and plant community composition (low diversity…3 spp.; high diversity…12 spp.)

**Parameter/**	**Low plant diversity**		**High plant diversity**	
**Plant species**	**-Ew**	**+Ew**	**-Ew**	**+Ew**
Maximum leaf length				
*A. elatius*	54.1 ± 4.1a	54.5 ± 2.6a	59.9 ± 1.3a	57.8 ± 2.3a
*P. vulgaris*	6.4 ± 0.7b	5.7 ± 0.6b	7.5 ± 0.5a	8.5 ± 0.4a
*T. pratense*	23.6 ± 1.3c	20.2 ± 1.5c	30.4 ± 1.2a	27.4 ± 1.0b
Number of tillers				
*A. elatius*	10.7 ± 1.1a	11.6 ± 1.3a	15.3 ± 2.2a	11.7 ± 1.1a
*P. vulgaris*	6.1 ± 0.9b	6.7 ± 0.8b	9.6 ± 1.4a	8.8 ± 0.8a
*T. pratense*	7.4 ± 0.6c	6.1 ± 0.4b	9.9 ± 1.0a	8.5 ± 1.1a

Different letters after values refer to significant differences within a species (Tukey-Tests, p < 0.05). Means ± SE, n = 6.

**Table 3:** ANOVA results for the effects of earthworms and plant community composition on plant growth, plant quality and slug herbivory

	**Earthworms**		**Plant community comp.**		**Ew x Pl.cc.**	
**Variable**	**F**	**P**	**F**	**P**	**F**	**P**
***Plant growth***						
**Focus spp. leaf length** (cm)	0.030	0.863	2.307	0.130	0.056	0.813
*A. elatius*	0.092	0.763	2.819	0.098	0.208	0.650
*P. vulgaris*	0.048	0.828	**12.106**	**0.001**	2.438	0.124
*T. pratense*	**6.181**	**0.016**	**28.918**	**<0.001**	0.028	0.866
**Focus spp. tiller numbers** (funct. gr.^-1^)	1.425	0.234	**12.396**	**0.001**	1.643	0.201
*A. elatius*	0.815	0.370	2.481	0.120	2.364	0.129
*P. vulgaris*	0.014	0.906	**7.704**	**0.007**	0.507	0.479
*T. pratense*	2.700	0.105	**9.094**	**0.004**	**0.001**	**0.003**
**Focus plants shoot biomass**	1.119	0.290	**4.012**	**0.045**	2.200	0.138
*A. elatius* shoot mass	**4.624**	**0.032**	**6.184**	**0.013**	**4.948**	**0.027**
*P. vulgaris* shoot mass	0.629	0.428	0.187	0.665	0.163	0.687
*T. pratense* shoot mass	0.009	0.926	**9.457**	**0.002**	0.006	0.936
**Total plant biomass** (g mesoc.^-1^)	3.375	0.073	**11.332**	**0.002**	**5.246**	**0.027**
Total shoot biomass	2.511	0.120	**10.986**	**0.002**	**5.751**	**0.021**
Total root biomass	2.770	0.103	**4.934**	**0.032**	1.281	0.264
Total grass shoots	0.344	0.560	**36.226**	**<0.001**	2.302	0.136
Total forb shoots	2.744	0.105	**133.920**	**<0.001**	**5.040**	**0.033**
Total legume shoots	0.891	0.350	0.115	0.736	0.304	0.584
***Plant quality***						
**Total plant C**_**tot **_**(%)**	0.096	0.758	0.114	0.736	0.004	0.949
*A. elatius* C_tot_	3.229	0.080	1.800	0.187	0.003	0.955
*P. vulgaris* C_tot_	0.001	0.988	0.018	0.893	0.010	0.922
*T. pratense* C_tot_	1.467	0.232	0.073	0.789	1.416	0.240
**Total plant N**_**tot **_**(%)**	**7.022**	**0.009**	0.215	0.643	0.159	0.691
*A. elatius* N_tot_	**5.728**	**0.021**	0.127	0.723	0.056	0.814
*P. vulgaris* N_tot_	**5.855**	**0.017**	2.070	0.160	0.098	0.756
*T. pratense* N_tot_	**5.604**	**0.022**	0.121	0.730	3.394	0.072
**Total plant C:N ratio**	**6.324**	**0.013**	0.232	0.631	0.045	0.833
*A. elatius* C:N ratio	**5.560**	**0.023**	0.001	0.985	0.095	0.756
*P. vulgaris* C:N ratio	0.360	0.553	1.484	0.232	0.073	0.789
*T. pratense* C:N ratio	**5.078**	**0.029**	0.001	0.992	2.561	0.117
***Slug herbivory***						
**No. damaged leaves after 3 days**	0.406	0.531	2.075	0.165	0.002	0.965
*P. vulgaris* leaves damaged	0.006	0.939	**4.531**	**0.041**	0.028	0.869
*T. pratense* leaves damaged	0.138	0.714	**5.248**	**0.033**	0.138	0.714
**No. damaged leaves after 6 days**	**7.667**	**0.012**	0.754	0.395	0.087	0.771
*P. vulgaris* leaves damaged	2.621	0.121	**11.346**	**0.003**	1.555	0.227
*T. pratense* leaves damaged	**4.961**	**0.038**	**24.719**	**<0.001**	**4.613**	**0.044**
**Total leaf area eaten mesocosm**^**-1**^	0.754	0.387	**10.371**	**0.002**	3.432	0.066
*P. vulgaris* leaf area eaten	0.374	0.543	**5.500**	**0.023**	1.750	0.191
*T. pratense* leaf area eaten	0.606	0.440	**6.803**	**0.012**	0.010	0.922

P-values after sequential Bonferroni corrrections.

**Figure 1:**
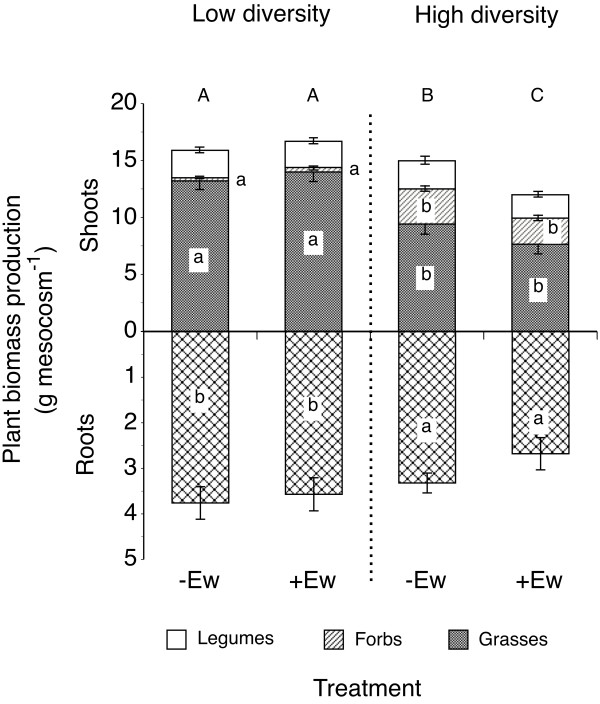
**Biomass production of grasses, non-leguminous forbs and leguminous forbs in mesocosms planted with different community composition (low diversity - 3 spp.; high diversity - 12 spp.) without (−Ew) or with earthworms (+Ew).** Only total roots are shown as they could not be assigned to certain functional groups. Different lower case letters denote significant differences among functional groups (Tukey, P < 0.05); different capital letters denote differences of total shoot mass. Means ± SE, n = 6.

Generally, earthworms directly affected *A. elatius* shoot mass (Table 3). However, plant community composition significantly increased total plant biomass, mainly due to an increased root mass at the cost of a significantly decreased shoot mass (Figure 1, Table 3). Response of shoot biomass production to plant community composition varied between plant functional groups with significantly decreased shoot mass in plant communities containing more plant species and significantly increased shoot mass of forbs (Figure 1, Table 3).

Neither earthworms nor plant community composition affected shoot total carbon concentration (Table 3, Table 4). Total plant N and N of focus species was significantly increased by earthworms; plant community composition had no effect on plant N (Table 3, Table 4). Consequently leaf C:N ratios were significantly lower when earthworms were present but unaffected by plant community composition (Table 3, Table 4).

**Table 4:** Leaf C, N and C:N ratios in response to earthworms (−Ew…no earthworms, +Ew…earthworm addition) and plant community composition (low diversity…3 spp.; high diversity…12 spp.)

**Parameter/**	**Low plant diversity**		**High plant diversity**	
**Funct. group**	**-Ew**	**+Ew**	**-Ew**	**+Ew**
C_tot_				
*A. elatius*	41.6 ± 0.3a	40.5 ± 0.4a	40.8 ± 0.5a	39.7 ± 1.0a
*P. vulgaris*	38.4 ± 1.0a	38.5 ± 0.5a	38.6 ± 0.1a	38.5 ± 0.8a
*T. pratense*	40.9 ± 0.3a	40.9 ± 0.3a	40.6 ± 0.3a	41.3 ± 0.4a
N_tot_				
*A. elatius*	2.9 ± 0.2b	3.5 ± 0.3a	2.8 ± 0.2b	3.4 ± 0.3a
*P. vulgaris*	2.7 ± 0.1b	3.1 ± 0.1a	2.9 ± 0.1b	3.4 ± 0.3a
*T. pratense*	3.0 ± 0.1ab	3.1 ± 0.2ab	2.8 ± 0.1c	3.3 ± 0.1a
C:N ratio				
*A. elatius*	15.9 ± 1.5a	12.3 ± 1.1b	15.3 ± 1.2a	12.7 ± 1.3b
*P. vulgaris*	14.3 ± 0.7a	12.8 ± 0.5b	13.9 ± 0.6a	11.5 ± 1.2b
*T. pratense*	13.9 ± 0.5a	13.5 ± 0.4a	14.7 ± 0.6a	12.7 ± 0.5b

Different letters after values refer to significant differences within a species (Tukey-Tests, p < 0.05). Means ± SE, n = 6.

**Figure 2:**
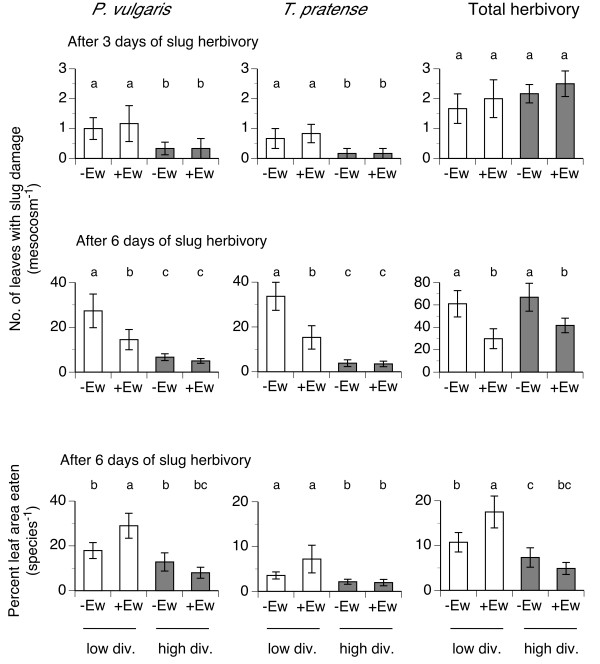
**Slug herbivory on the forb *P. vulgaris*, the legume *T. pratense *and to total plant community measured three days (uppermost graphs) and six days after slug introduction (middle graphs and bottom graphs).** Different letters denote significant differences among treatments within each plant species (Tukey, P < 0.05). Means ± SE, n = 6.

Three days after slug introduction, significantly less leaves of *P. vulgaris* and *T. pratense* were damaged in plant communities containing twelve compared to three species; earthworms had no effect on slug damage after three days; overall herbivory after three days was unaffected by earthworms or plant community composition (Figure 2, Table 3). Six days after slug introduction, significantly less leaves and leaf area of *P. vulgaris* and *T. pratense* were consumed in plant communities containing twelve compared to three species; earthworms significantly reduced number of damaged leaves only in low diversity mesocosms and had no effect in high diversity mesocosms (Figure 2). Total number of damaged leaves was significantly lower in +Ew treatments but unaffected by plant community composition. Percent leaf area consumed (total species and focus species) was significantly lower in plant communities containing twelve species (Figure 2, Table 3). Earthworms increased eaten leaf area only for *P. vulgaris* and total plant community in communities containing only three plant species (Figure 2). Damage was not correlated to plant C, N contents or C-to-N ratio (data not shown).

## Discussion

Our results demonstrate that herbivory by the most important invasive slug species in Europe is influenced by earthworms, and that this influence varies with the species composition of plant communities. Although, plant communities with different numbers of plant species were similarly attacked by slugs (measured as number of damaged leaves), slugs consumed significantly less leaf area in plant communities containing twelve compared to three species. Leaf consumption of slugs was increased by earthworms in plant communities with fewer plant species but unaffected by earthworms in communities with more plant species. In the literature, results on effects of plant diversity on herbivory are heterogeneous ranging from no effect in herbivore damage of individual plants across a gradient of one to 60 plant species [46] to a decreased herbivory with increasing plant species richness [47]. For the current study it is important to note that due to the design of the experiment we cannot distinguish between the effects of species diversity *per se* and the effects of species composition of the compared plant communities [48].

### Earthworm effects on slug herbivory

Our first hypothesis that earthworms would differentially affect the growth and nutrient content of plant species that will consequently also alter the food choice of slugs was partly confirmed. While earthworms did not affect leaf length and number of tillers of three focus species, they changed the structure of plant communities by further reducing total aboveground biomass production in plant communities containing more plant species that already showed less productivity than plant communities containing three species only. Thus, reduced slug herbivory in communities with more plant species could also be the consequence of a greater choice of food sources and a less denser plant community structure, although other studies investigating different slug and plant species showed little influence of different food quantity on slug herbivory [49,50].

Our second hypothesis that effects of earthworms will vary with plant community composition as communities containing more species commonly show a more complete resource use and earthworm-induced nutrient benefits would be less effective was also partly confirmed. Our data also suggest that the significantly higher root mass in stands with more plant species might have stimulated earthworm activity [18,51] and thus increased their impact on slug herbivory. Earthworms increased N content of the three focus plant species regardless of the number of plant species present in plant communities, indicating a better nutrient availability of these plant species due to earthworm activity. Less diverse stands with a denser vegetation (more aboveground biomass) probably also had a microclimate more favourable for slugs [52]. The finding that earthworms reduced slug attacks regardless of plant community composition suggests that earthworm-induced changes in the chemical defense ability of plants [42,53] is independent of plant community composition, however the possible role of secondary metabolites and defensive compounds on slug herbivory remains to be further investigated. Explanations for the stimulated leaf consumption in plant communities containing fewer species could be that (i) slugs were initially browsing (tasting) across available plant species causing lesions on leaves [54] and plants in mesocosms containing earthworms tasted less well or were better defended (e.g. by N-rich defense compounds), (ii) consumption in low diversity mesocosms was higher because more shoot mass was available known to influence slug herbivory [55].

Previous work has shown that earthworm biomass and activity is influenced by plant diversity [18,19,21] and that earthworms themselves can influence plant diversity and growth [22,26,56]. The current results further indicate that the susceptibility of plant communities to herbivore attack is driven by a complex interaction between belowground detritivores, the community composition and productivity of grassland plant communities and the feeding behaviour of this generalist herbivore. Our results are in line with findings showing that earthworm impacts were of less importance in high diverse plant communities [11], likely due to high plant structural complexity. The former study also indicates that earthworms modulate the diversity-invasibility relationship and the stability of grassland plant communities. The current experimental setup precludes statements on invasibility in a strict sense as we added slugs, however, if we translate our findings to field conditions it could mean that managed grassland communities containing less plant species are more prone to herbivory by this invasive slug species and that earthworms may even increase this risk.

We observed a marked decline of earthworm numbers and biomass during the course of the experiment. Such declines are frequently observed in earthworm laboratory studies, especially when experiments lasted several months [26,57]. Based on our observation that earthworm activity changed little until the end of the experiment we assume that (i) the decline of earthworm numbers and weight loss occurred mainly within the last three days before harvest when we discontinued watering to facilitate the harvesting process, (ii) some worms might have been overlooked during the destructive harvest in the 20 l mesocosms.

### Consequences for plant communities

Slugs avoided the grass and preferred forbs and legumes in both plant communities which would lead towards more grassy communities in the long run. According to the current results, this process is expected to be enhanced as earthworms increase slug damage in communities containing less species. Although our plant communities were planted with same densities, plant functional group composition showed a shift towards significantly more grass and less forb biomass when only three plant species were present and a more balanced composition between functional groups when twelve plant species were present. This is also in line with the finding that earthworms increased leaf area consumed by slugs only at low diversity because especially the biomass production of the grass species was stimulated by earthworms [12,22]. Our third hypothesis that more plant species in a community will lead to reduced herbivory was confirmed. However, to what extent the decreased shoot biomass in these communities contributed to this feeding pattern demands further investigation.

Our results show a contradictory impact of earthworms on slug damage after three and six days of exposure. We assume that this depends merely on the fact that slugs became acquainted with different food sources [54]. It has been shown that earthworms influence the resistance of plant communities against plant invaders and this effect varied with plant diversity and with time [11,20], possibly by altering the diversity-stability relationship. The current study shows that earthworms also affect herbivore invaders and that this effect varies with the composition of plant communities. So far only a few studies have confirmed the predicted decrease in herbivore damage in more diverse plant communities [47,58,59].

Although planting densities were the same under both plant community compositions, more plant species in a community led to significantly more tillers and greater leaf lengths of model forb *P. vulgaris* and model legume *T. pratense*, whereas the model grass *A. elatius* remained unaffected by earthworms or plant community composition. We explain this by a greater competitive ability of these plants and a more complete resource use of these communities. In contrast to other studies earthworms did not promote biomass production of individual plant species [22,24], moreover at high diversity lower biomass was produced. The finding that low diversity stands have a higher biomass production than high diversity stands is well reflected in the literature [25] and can be attributed to niche-exploitation. There was a significant shift in biomass allocation from shoots to roots between plant communities containing three vs. twelve plant species suggesting a higher competition for available resources in communities containing more plant species. A similar pattern with reduced shoot biomass and N uptake in the presence of earthworms of the legume *T. repens* was reported earlier [24]. Earthworms have been shown to enhance the growth of grasses under short-term experimental conditions while legumes are more likely to respond only in the long-term [22,27,60]. But even if there was an increased N mineralization through earthworms it would mainly benefit grasses anyway [12]. The majority of studies showed growth stimulation by earthworms [28,61], however many of these studies investigated single plants only, while earthworm effects conducted in plant communities frequently show little or no earthworm effect on biomass production [27].

## Conclusions

Taken together, our results show that aboveground invasive generalist herbivores can be influenced by belowground detritivores and that this influence can depend on the composition of the plant community. Our results suggest that communities containing more plant species are less prone to be attacked by this slug herbivore; in plant communities containing less plant species earthworms can make leaves even more attractive for slugs. In order to fully understand plant species interactions and ecosystem functioning, these belowground-aboveground linkages [62,63] need to be considered more widely.

## Competing interests

All authors declare that they have no competing interests.

## Authors’ contributions

All authors contributed to data collection and/or analysis of project results. JGZ, TF and TD wrote the majority of the paper with contributions from the co-authors. All authors have read and approved the final manuscript.
